# Nitrosative/Oxidative Stress Conditions Regulate Thioredoxin-Interacting Protein (TXNIP) Expression and Thioredoxin-1 (TRX-1) Nuclear Localization

**DOI:** 10.1371/journal.pone.0084588

**Published:** 2013-12-20

**Authors:** Fernando Toshio Ogata, Wagner Luiz Batista, Adriano Sartori, Tarsis Ferreira Gesteira, Hiroshi Masutani, Roberto Jun Arai, Junji Yodoi, Arnold Stern, Hugo Pequeno Monteiro

**Affiliations:** 1 Departamento de Bioquímica/Biologia Molecular and Center for Cellular and Molecular Therapy – CTCMol – Universidade Federal de São Paulo, São Paulo, Brazil; 2 Departamento de Ciências Biológicas, Universidade Federal de São Paulo, Campus Diadema, São Paulo, Brazil; 3 Department of Biological Responses, Kyoto University, Kyoto, Japan; 4 Department of Pharmacology, New York University School of Medicine, New York, New York, United States of America; 5 Instituto do Câncer do Estado de São Paulo, Faculdade de Medicina da Universidade de São Paulo, São Paulo, Brazil; University of Sassari, Italy

## Abstract

Thioredoxin (TRX-1) is a multifunctional protein that controls the redox status of other proteins. TRX-1 can be found in the extracellular milieu, cytoplasm and nucleus, and it has distinct functions in each environment. Previously, we studied the intracellular localization of TRX-1 and its relationship with the activation of the p21Ras - ERK1/2 MAP Kinases signaling pathway. In situations where this pathway was activated by stress conditions evoked by a nitrosothiol, S-nitroso-N-acetylpenicillamine (SNAP), TRX-1 accumulated in the nuclear compartment due to nitrosylation of p21Ras and activation of downstream ERK1/2 MAP kinases. Presently, we demonstrate that ERK1/2 MAP Kinases activation and spatial distribution within cells trigger TRX-1 nuclear translocation through down-regulation of the physiological inhibitor of TRX-1, Thioredoxin Interacting Protein (TXNIP). Once activated by the oxidants, SNAP and H_2_O_2_, the ERK1/2 MAP kinases migrate to the nucleus. This is correlated with down-regulation of TXNIP. In the presence of the MEK inhibitors (PD98059 or UO126), or in cells transfected with the Protein Enriched in Astrocytes (PEA-15), a cytoplasmic anchor of ERK1/2 MAP kinases, TRX-1 nuclear migration and TXNIP down-regulation are no longer observed in cells exposed to oxidants. On the other hand, over-expression of TXNIP abolishes nuclear migration of TRX-1 under nitrosative/oxidative stress conditions, whereas gene silencing of TXNIP facilitates nuclear migration even in the absence of stress conditions. Studies based on the TXNIP promoter support this regulation. In conclusion, changes in TRX-1 compartmentalization under nitrosative/oxidative stress conditions are dependent on the expression levels of TXNIP, which are regulated by cellular compartmentalization and activation of the ERK1/2 MAP kinases.

## Introduction

Thioredoxin-1 (TRX-1), a 12 kDa protein with conserved cysteines at its redox active site, plays major roles in cellular redox balance and signaling by maintaining a reducing intracellular microenvironment [1]. Intracellular location of TRX-1 will determine its signaling properties. TRX-1 is encountered in the extracellular compartment, in the cytoplasm, and in the nucleus where it regulates the activities of several transcription factors [2]. In addition to the redox regulation of transcription factors, TRX-1 nuclear localization was associated with cell survival [3]. 

Nerve growth factor a major survival factor of sympathetic neurons induced TRX-1 nuclear translocation in rat pheochromocytoma PC12 cells. PD98059, an inhibitor of MEK, which phosphorylates and activates the ERK1/2 MAP kinases, suppressed nuclear translocation of TRX-1 and neuron survival [4]. Our previous work demonstrated that in HeLa cells exposed to increasing concentrations of the low molecular weight nitrosothiol S-nitroso-N-acetylpenicillamine (SNAP), TRX-1 nuclear migration was stimulated. The SNAP-induced TRX-1 nuclear migration was directly associated with the activation of the p21Ras – ERK1/2 MAP kinases survival signaling pathway. Inhibition of p21Ras or MEK in HeLa cells prevented TRX-1 nuclear migration and increased the rate of cell death [3].

 Thioredoxin-interacting protein (Txnip) or Thioredoxin Binding Protein 2 (TBP-2) was originally described as a vitamin-D3-upregulated protein (VDUP-1) [5]. Subsequent studies conducted by Yodoi and coworkers demonstrated *in vitro* and *in vivo* the association of TRX-1 with TXNIP. TXNIP binds to reduced TRX-1 but not to oxidized TRX-1 nor to mutant TRX-1 in which two redox active cysteine residues are substituted by serine. The catalytic center of TRX-1 seems to be important for the interaction [6]. Being characterized as a negative regulator of TRX-1 functions, TXNIP was implicated as a suppressor of TRX-1-mediated pro-survival signaling pathways and it migrates to the nucleus via importin α1 [7].

 Wang et al. demonstrated that adenovirus-mediated over-expression of TXNIP suppressed TRX-1 activity in rat cardiomyocytes. Suppression of TRX-1 activity induced cardiomyocyte apoptosis [8]. On the other hand, TXNIP down-regulation by H_2_O_2_ or by exposure of cells to exogenous S-nitrosoglutathione led to an increase in TRX-1 activity [8,9]. TRX-1 nuclear migration in signaling pathways is associated with increasing cell survival, tumor development and metastasis has been described [4,10,11]. TRX-1 migration to the nucleus apparently does not occur via classical nuclear localization signal [2].

 The aim of the present study is to investigate the role of TXNIP on TRX-1 nuclear migration induced by nitrosative/oxidative stress conditions; the participation of the ERK1/2 MAP kinases as mediators of this process is also investigated. Under nitrosative/oxidative stress conditions, ERK1/2 MAP kinases activation and their nuclear migration down-regulate TXNIP expression, promoting TRX-1 nuclear migration.

## Materials and Methods

### Materials and Reagents

Hydrogen peroxide (H_2_O_2_), (±)-S-Nitroso-N-acetylpenicillamine (SNAP) 4-amino-5-methylamino- 2′,7′-difluorofluorescein diacetate (DAF-FM), UO126 and PD98059, selective and cell permeable inhibitors of MEK, were purchased from Calbiochem (La Jolla, CA). 3-(4,5-Dimethylthiazol-2-yl)-2,5-diphenyltetrazolium (MTT) and anti-β-actin mouse monoclonal antibody were obtained from Sigma-Aldrich (St. Louis, MO). Anti-phospho ERK 1/2 MAP Kinases, anti-ERK 1/2 MAP Kinases, anti-Akt rabbit polyclonal antibodies, and anti-phosho Akt mouse monoclonal antibody were purchased from Cell Signaling Technology (Boston, MA). Anti-human TRX-1 monoclonal antibody (11 mAB) was provided by Redox Bioscience (Kyoto, Japan). Anti-human VDUP (TXNIP) mouse monoclonal antibody was provided by Santa Cruz Biotech (Dallas, TX). Lactate Dehydrogenase (LDH) detection kit was from Roche Applied Science (Penzberg, Germany). Conventional PCR and real-time PCR primers were purchased from IDT (Coralville, IA). Rhodamine-conjugated goat anti-mouse IgG and the Super Signal chemiluminescence detection system purchased from Thermo-Pierce (Rockford, IL). Restriction enzymes were purchased from Fermentas (Vilnius, Lithuania). Minimun essential media (MEM) was purchased from Invitrogen (Grand Island, NY). Fetal bovine serum was purchased from Cultilab (São Paulo, Brazil).

### Mammalian Cell Culture and Transfections

The human cervical carcinoma cell line – HeLa – obtained from American Type Culture Collection was cultured in Minimum Essential Medium (MEM) supplemented with 10% fetal bovine serum (FBS), penicillin (100 U/ml) and streptomycin (100 µg/ml) at 37 °C, 5% CO_2_. The expression vector pcDNA3_PEA-15 was kindly provided by Professor Joe Ramos (Hawaii, USA). TXNIP gene cloning was achieved by PCR of total cDNA with specific primers: TXNIP-5´_EcoRI GAA TTC ATG GTG ATG TTC AAG AAG ATC and TXNIP-3´_SmaI GGG CCC TCA CTG CAC ATT GTT. The amplicon was cloned in the pGEM (Promega – Madison, WI) cloning vector and the product of digestion was sub-cloned in the expression vector pcDNA3 (Invitrogen – Grand Island, NY). The same procedure was used to obtain the promoter region of TXNIP. The pair of primers used for this was: promoter TXNIP-5´_AseI ATT AAT TAC TTC AGT CAG AGT T and promoter TXNIP-3´_NheI GCT AGC GAA GTC AGA GAA AAA G. But instead this amplicon was sub-cloned excising the CMV promoter region from pEGFP-C1 (Clonteh – Mountain View, CA) using AseI and NheI enzymes.

TXNIP gene silencing expression vector (shRNA TXNIP) was constructed following the manual provided by the manufacturer (Invitrogen p*Silencer*™ 4.1-CMV neo Kit AM5779). Briefly, the oligonucleotides sequence was obtained in [12] and was synthesized by IDT DNA Technologies according to manufacturer's specifications (Top TXNIP 5´- GAT CCA CAG ACT TCG GAG TAC CTG TTC AAG AGA GAC AGG TAC TCC GAA GTC TGT TTA -3´ and Bottom TXNIP 5´- AGC TTA AAC AGA CTT CGG AGT ACC TGT CTC TTG AAC AGG TAC TCC GAA GTC TGT G -3´). Oligonucleotides were dilute in water and annealed by thermal variation. A double strand sequence was then ligated into the vector from the kit and incubated for 14 h at room temperature with T4 DNA ligase.

Plasmids were transfected into cells by using FuGENE HD Transfection Reagent (Roche Applied Science - Penzberg, Germany) according to the manufacturer´s instructions. 

### Determination of intracellular levels of ^·^NO

HeLa cells were cultured in MEM supplemented with 10% FBS for 24 h at 5% of CO_2_ and 37° C. After this period cells had their medium replaced by MEM without serum. Cells were pre-incubated with 5 µM DAF-FM for 30 min, prior to stimuli with SNAP (0.5 mM for two h at 37°C). After incubation, cells were trypsinized and their fluorescence content measured by flow cytometry. Data were analyzed using the Cyflogic software (www.cyflogic.com).

### Glutathione Peroxidase and Catalase activity measurements

Cells were lysed in lysis buffer (50 mM Tris/HCl, pH 7.4; 1 mM EDTA; 500 mM PMSF). Cell lysates were incubated on ice for 10 min and centrifuged at 10,000 rpm for 10 min. Protein concentrations of supernatants were determined as previously described [13] and 20 µg of protein from cell lysates were used to determine the activities of Glutathione peroxidase (GPx) and Catalase.

GPx total activity was determined using tert-butyl hydroperoxide (1.2 mM) as substrate. The absorbance at 340 nm was monitored for 5 min in 100 mM phosphate buffer, pH 7.0, EDTA 1 mM at 37°C. One unit of activity is defined as the amount of protein capable of oxidizing 1 mM NADPH per minute and is expressed as U/mg of protein [14]. 

Catalase activity was determined from total cell lysates by monitoring the exponential decay of the absorbance at 240 nm (A_240nm_ = 39.4 M^-1^ · cm^-1^) of a solution of 10 mM H_2_O_2_ in 100 mM phosphate buffer, pH 7.0 at 25°C. Results are expressed as U/mg proteins [15].

### Cell viability assay

1x10^5^ cells were plated in MEM in 96 wells plates. The plate was incubated overnight at 5% of CO_2_ and 37° C. Medium was replaced for MEM supplemented with 10% FBS and H_2_O_2_ or SNAP in a range of concentrations (0.1 - 1.0 mM). After 2 h medium was replaced for fresh MEM supplemented with 10% FBS and cells were let in the incubator (5% of CO_2_ and 37° C) for 24 h. Finally, MTT solution was prepared to a final concentration of 5mg/ml in PBS and 50 μl of this stock solution were added to each well to a final volume of 200 μl. Plates were incubated for additional 4 h at 37° C. Solution was discarded and the precipitate was dissolved in dimethyl sulfoxide (200 μl). The absorbance of the dimethyl sulfoxide-based solution was measured at 570nm.

### Real-Time PCR

Total RNA were treated with DNase (Promega – Madison, WI) according to the manufacturer’s instructions and an aliquot from the treated RNA was reverse-transcribed to cDNA using the SuperScript First-Strand Synthesis System for RT-PCR (Invitrogen – Grand Island, NY). Quantitative real-time (qRT) - PCRs were performed using SYBR Green PCR Master Mix (Applied Biosystems - Foster City, CA) in a Applied Biosystems 7500 Real-Time PCR System (Applied Biosystems - Foster City, CA). The primers sequences optimized for qRT-PCR were as follows: TRX-5' TGG TGA AGC AGA TCG AGA GCA AGA and TRX-3' ACC ACG TGG CTG AGA AGT CAA CTA. TXNIP-5` ACT CGT GTC AAA GCC GTT AGG and TXNIP-3` TCC CTG CAT CCA AAG CAC TT. GFP-5' TGA CCC TGA AGT TCA TCT GCA CCA and GFP-3' TCT TGT AGT TGC CGT CGT CCT TGA. RPL13A 5` CCT GGA GGA GAA GAG GAA AGA GA and RPL13A 3` TTG AGG ACC TCT GTG TAT TTG TCA A. Beta-5´ CAT CTG CTG GAA GGT GGA CA and Beta-3´ GCT CCT CCT GAG CGC AAG. qRT-PCR assays were performed in triplicates. The parameters used for the PCR were 50 °C for 2 min, 95 °C for 10 min, 40 cycles of 95 °C for 15 s, and 60 °C for 1 min. The relative expression ratio (experimental/control) was determined based on the 2^-ΔΔCt^ method [16]. 

### Indirect immunofluorescence cell staining

Brieﬂy, cells were ﬁxed with 3.7% paraformaldehyde in PBS containing 10% fetal bovine serum, for 20 min at room temperature, followed by permeabilization for 10 min using 0.2% (w/v) Triton X-100 in PBS. After incubation with a mouse monoclonal antibody against human TRX-1 or rabbit polyclonal antibody against phospho-ERK 1/2 MAP kinases for 1 hour at room temperature, the slides were incubated for 1 hour with a goat anti-mouse IgG secondary antibody conjugated with rhodamine (Pierce – Rockford, IL) or with a goat anti-rabbit IgG antibody conjugated with fluorescein isothiocyanate (Invitrogen – Grand Island, NY). Slides were also incubated with 4',6-Diamidine-2'-phenylindole dihydrochloride (DAPI) for nuclear labeling (Boehringer–Manheimm, Germany). Slides with stained cells were analyzed in a LSM 510 confocal microscope (Carl Zeiss, Germany). 

### Time-lapse live confocal microscopy

Cells were plated at 2–4x10^5^ per plate containing a 25-mm glass coverslip. They were stably transfected with expression vectors pcDNA_PEA-15 and pcDNA3_TXNIP, and transiently transfected prior to confocal microscopy with pEGFP-C1_Trx-1 using FuGENE HD reagent (Roche Applied Science - Penzberg, Germany). The experiment was carried out after 18 h of transfection. The coverslips were kept heated (37°C) and live cells were imaged with a Zeiss 510 inverted laser-scanning confocal microscope LSM-510 NLO (Carl Zeiss, Germany). Cells were imaged as previously described [17]. H_2_O_2_ was added to the media to a final concentration of 0.5 mM. Multiple images (at least 5–6 slices of Z= 0.45 (µm) were captured before and then every 3 min after the induction period for a total of 120 min.

### Preparation of nuclear extracts from HeLa cells

Nuclear fractions were obtained as follows: cytoplasmic fractions were extracted with buffer containing: 10 mM Tris pH 7.4, 10 mM NaCl, 3 mM MgCl_2_, 0.5% NP-40, 0.5 mM PMSF, 10 µg/ml, and 10 µg/ml aprotinin. The nucleus was separated in 1.0 mM sucrose buffer (centrifuged at 1,500g) and the nuclear content was extracted with buffer containing: 200 mM NaCl, 10 mM Hepes pH 7.9, 1.5 mM MgCl_2_, 0.1 mM EDTA, 5% glycerol, 0.5 mM PMSF, 10 µg/ml leupeptin, and 10 µg/ml aprotinin. Cytoplasmic and nuclear fractions were submitted to LDH detection according to manufacturer´s instructions to estimate cytoplasmic contamination in nuclear extracts. The indicated values show the ratio of nuclear and cytoplasmic LDH per mg of protein. 

### Western blot analysis for TRX-1, TXNIP, Akt, and ERK1/2 MAP Kinases

At each experimental point, cells were lysed in 500 µL lysis buffer (20 mM Hepes, 150 mM NaCl, 1.5 mM MgCl_2_, 1 mM EGTA, 10% glycerol, 1% Triton X-100, 1 μg/ml aprotinin, 1 μg/ml leupeptin, and 1 mM PMSF, pH 7.5) supplemented with phosphatases inhibitors. Total cell lysates (50 µg/lane) were resolved in 12% SDS–PAGE gels and blotted onto Polyvinylidene fluoride (Immobilon-P - PVDF) Millipore (Billerica, MA) sheets. Blots were probed using specific antibodies against TRX-1, TXNIP, the ERK1/2 MAP Kinases, phospho-ERK1/2 MAP Kinases, Akt, phospho-Akt, and β-actin used as a protein loading control. After incubation with appropriated HRP-conjugated secondary antibodies, immunoblots were developed using the Super Signal system (Thermo-Pierce – Rockford, IL) and digitally registered on LAS-4000 (Fujifilm – Tokyo, Japan).

### Statistical analysis

Results are expressed as means ± S.D. Results are the mean of at least three separate experiments in each group. Statistical testing involved a 2-tailed paired student t-test or one way ANOVA.

## Results

### Oxidative and nitrosative stress conditions established in HeLa cells exposed to increasing concentrations of H_2_O_2_ and SNAP

**Figure 1:**
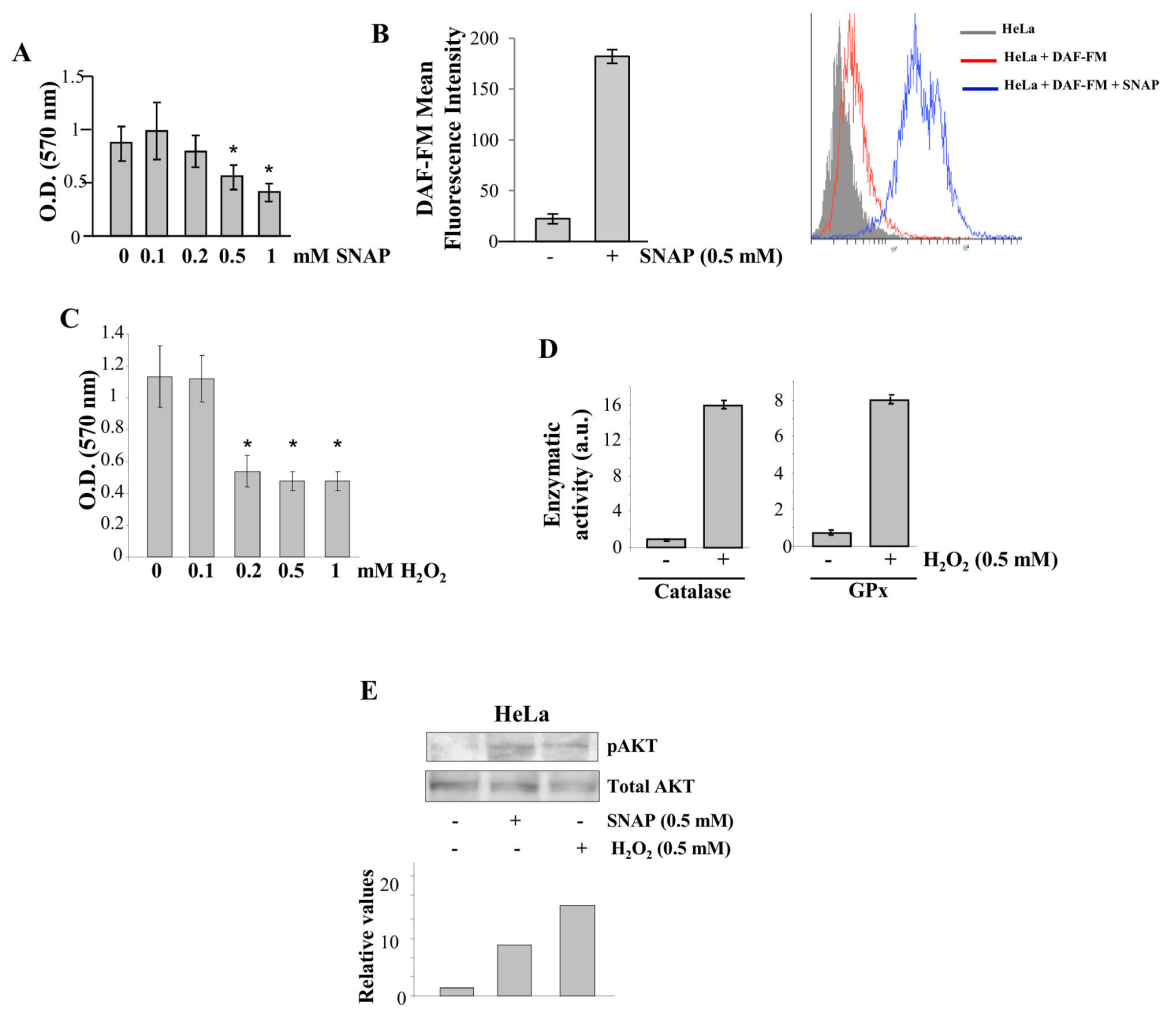
HeLa cells submitted to oxidative and nitrosative stress conditions. (**A**) HeLa cells were treated with increasing concentrations of SNAP (0 - 1.0 mM) during 2 h. After this period medium was replaced for complete medium (MEM supplemented with 10% FBS). Cell viability was determined 24 h later using the MTT assay as described in Materials and Methods. Values are reported in the bar graphs and expressed as means ± S.D. (n = 3, *p < 0.05). (**B**) Intracellular NO detection in HeLa cells after incubation with 0.5 mM SNAP for 2 h at 37°C. DAF fluorescence is directly related to NO generation by intracellular metabolism of SNAP and was determined by flow cytometry. (**C**) HeLa cells were treated with increasing concentrations of H_2_O_2_ (0 - 1.0 mM) during 2 h. After this period medium was replaced for complete medium (MEM supplemented with 10% FBS). Cell viability was determined 24 h later using the MTT assay as described in Materials and Methods. Values are reported in the bar graphs and expressed as means ± S.D. (n = 3, *p < 0.05). (**D**) Catalase and Glutathione Peroxidase enzymatic activity assays (described in Materials and Methods) were performed after exposure of HeLa cells to 0.5 mM H_2_O_2_ for 2 h at 37°C. (**E**) HeLa cells were exposed to 0.5 mM SNAP or to 0.5 mM H_2_O_2_ for 2 h at 37°C. After cell lysis, 50 μg total protein were subjected to Western blotting with a rabbit polyclonal anti-phospho-Akt antibody and with a mouse monoclonal anti-Akt antibody. Western blot results are representative of three independent experiments. Histogram represents the ratio between the densitometric values of the protein bands corresponding to the phosphorylated form of Akt and of Akt protein of one representative experiment (Lower panel).

Exposure of HeLa cells to increasing concentrations of the low molecular weight s-nitrosothiol, SNAP, leads to loss of cell viability (Figure 1A), which is a reflection of nitrosative stress conditions [18]. The concentration of 0.5 mM SNAP, which promoted ~ 50% loss of cell viability, was used in the subsequent experiments. As indicated by DAF-FM-derived fluorescence, the intracellular levels of ^·^NO significantly increased in HeLa cells after 2 h incubation with 0.5 mM SNAP. This was an indication that remaining viable cells were capable of metabolizing the intracellular fluorescent probe DAF-FM (Figure 1B).

Increasing loss of cell viability was observed after exposure of HeLa cells to concentrations of H_2_O_2_ greater than 0.1 mM (Figure 1C). The concentration of 0.5 mM H_2_O_2_, which caused ~ 60% loss of cell viability, was used in the subsequent experiments. Although, 0.5 mM H_2_O_2_ caused loss of cell viability, remaining viable HeLa cells exposed to these concentrations of H_2_O_2_ retained their ability to metabolize the oxidant. Catalase and GPx activities were stimulated in HeLa cells incubated with 0.5 mM H_2_O_2_ during 2 h at 37°C (Figure 1D). Exposure of HeLa cells to both stressors resulted in activation of Akt (Figure 1E), characterizing a survival response to the conditions imposed to them. 

### Stress induced TRX-1 sub cellular compartmentalization is associated with activation and localization of the ERK 1/2 MAP Kinases

**Figure 2:**
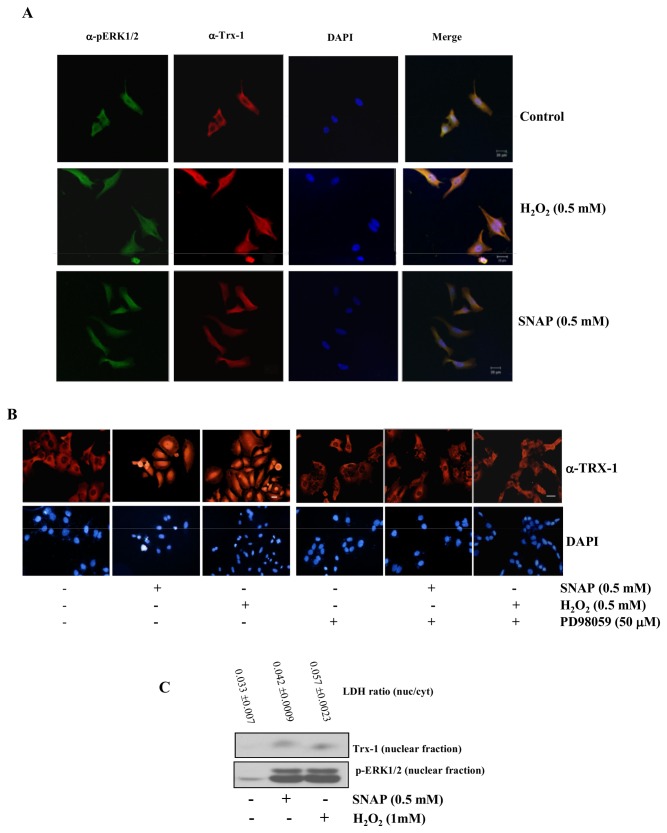
Confocal microscopy and western blot analysis to determine TRX-1 and phospho ERK 1/2 MAP Kinases sub-cellular location in wild-type HeLa cells. (**A**) Cells were treated with SNAP (0.5 mM), or H_2_O_2_ (0.5 mM) for 2 h at 37°C. Detection of TRX-1 and phospho-ERK 1/2 MAP Kinases was performed as described in Materials and Methods. Bar = 20µm. (**B**) Cells were treated with SNAP (0.5mM) or H_2_O_2_ (0.5 mM) for 2 h at 37°C. HeLa cells were pre-treated with 50μM PD98059, a MEK inhibitor, for 30 min and incubated with 0.5mM SNAP or 0.5 mM H_2_O_2_ for 2 h at 37°C. Detection of TRX-1 was performed on a fluorescence microscope as described in Materials and Methods. Images shown are representative from three independent experiments. Bar = 20µm. (**C**) HeLa cells were incubated with SNAP and H_2_O_2_ at the indicated concentrations for 2 h and nuclear extracts were subjected to Western blotting with the anti-human TRX-1 mouse monoclonal antibody and with the anti-human phospho-ERK1/2 MAP Kinases rabbit polyclonal antibody. Western blot results are representative of three independent experiments. Purity of nuclear fractions was estimated by LDH detection as described in Materials and Methods. Values are means ± S.D. of triplicate cultures.

The relationship between TRX-1 cellular compartmentalization and the activation/phosphorylation of the ERK1/2 MAP kinases was investigated using confocal microscopy. HeLa cells were incubated for 2 h at 37°C with SNAP (0.5 mM) or H_2_O_2_ (0.5 mM) and then labeled with the antibodies that recognize TRX-1 conjugated with fluorescein isothiocyanate and phospho-ERK1/2 MAP kinases conjugated with rhodamine. TRX-1 labeling superimposed with phosphorylated ERK1/2 labeling and with nuclear labeling (DAPI) in cells incubated with both stressors indicating that both proteins migrate to the nucleus (Figure 2A). Inhibiting phosphorylation of the ERK1/2 MAP kinases by pre-incubation of HeLa cells with MEK inhibitor PD98059 for 30 min, prior to 2 h exposure to the oxidants SNAP (0.5 mM) or H_2_O_2_ (0.5 mM), prevented stress-induced TRX-1 nuclear migration (Figure 2B). Identical results were obtained with another MEK inhibitor UO126 (Figure S1). 

Western blot analysis of nuclear extracts of HeLa cells exposed to SNAP or to H_2_O_2_ confirmed that both stressors induced phosphorylation of ERK1/2 MAP kinases and TRX-1 nuclear translocation (Figure 2C). These observations confirmed and extended our previous observations [3]. 

**Figure 3:**
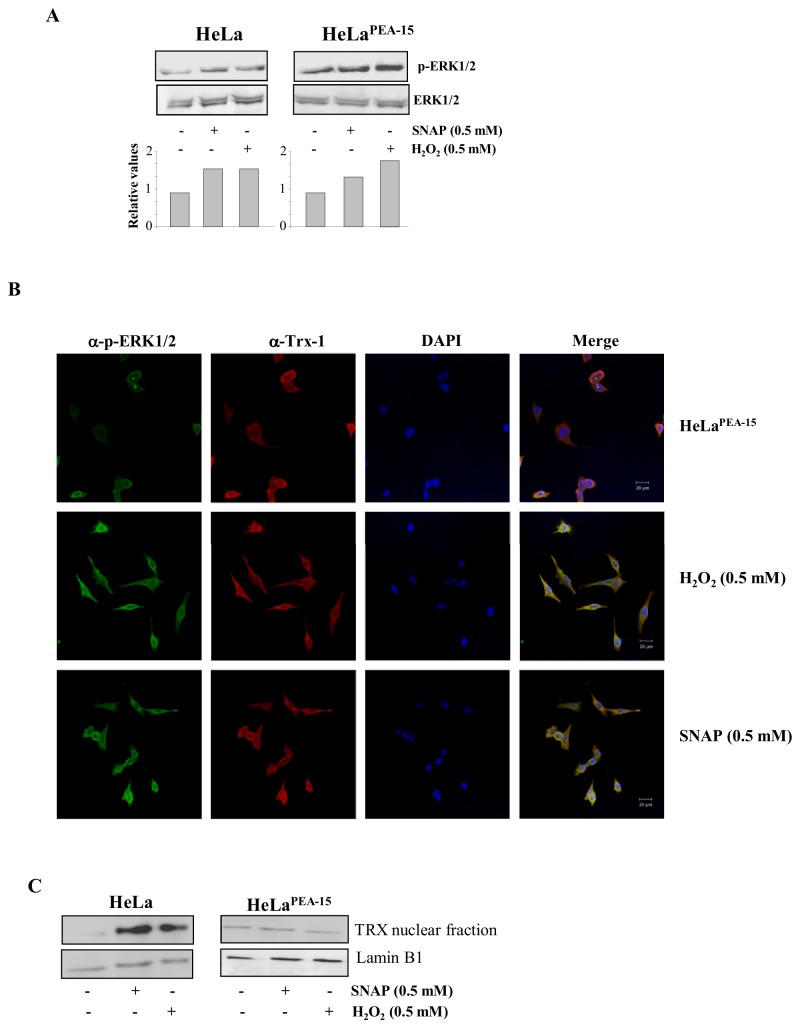
Stress-induced sub-cellular localization and activation of TRX-1 and phospho ERK 1/2 MAP Kinases in HeLa cells expressing PEA-15. (**A**) HeLa cells and HeLa cells expressing PEA-15 were incubated with SNAP (0.5 mM) or H_2_O_2_ (0.5 mM) for 2 h at 37°C. Cells were lysed and phosphorylation of the ERK1/2 MAP kinases was examined by western blotting. Western blot results are representative of four independent experiments. Histogram represents the ratio between the densitometric values of the protein bands corresponding to the phosphorylated form and of the total ERK 1/2 MAP kinases (Lower panel). (**B**) HeLa cells expressing PEA-15, were treated with SNAP (0.5 mM), or H_2_O_2_ (0.5 mM) for 2 h at 37°C. Sub-cellular localization of TRX-1 and phospho-ERK 1/2 MAP Kinases was performed as described in Materials and Methods. Bar = 20µm. (C) HeLa cells and HeLa cells expressing PEA-15 were incubated with SNAP (0.5 mM) or H_2_O_2_ (0.5 mM) for 2 h at 37°C. Nuclear extracts were subjected to Western blotting with the anti-human TRX-1 mouse monoclonal antibody. Western blot results are representative of three independent experiments.

HeLa cells permanently transfected with a plasmid containing the gene encoding the expression of the 15 kDa protein enriched in astrocytes (PEA-15) were used to analyze whether or not the ERK1/2 MAP kinases localization is relevant to stress-induced TRX-1 nuclear translocation. PEA-15 has been described as a cytoplasmic anchor for ERK1/2 MAP kinases [19]. H_2_O_2_ and SNAP stimulated phosphorylation of ERK1/2 MAP kinases in parental and in HeLa cells over expressing PEA-15, ruling out any spurious effect on ERK1/2 MAP kinases activities related to their anchorage in the cytoplasm (Figure 3A). 

Intracellular localization of TRX-1 and ERK1/2 MAP kinases was further investigated in HeLa cells expressing PEA-15 incubated with 0.5 mM SNAP or 0.5 mM H_2_O_2_. Under these conditions, nuclear migration of both TRX-1 and phosphorylated ERK1/2 MAP kinases was inhibited, resulting in co-localization of those proteins in the cytoplasm (Figure 3B). Western blot analysis for TRX-1 of the nuclear fractions of HeLa cells and HeLa cells expressing PEA-15 incubated with 0.5 mM SNAP or 0.5 mM H_2_O_2_, showed that TRX-1 nuclear migration occurred only in parental HeLa cells stimulated with both stressors (Figure 3C). Results strongly suggest that stress-induced TRX-1 nuclear migration is associated with simultaneous nuclear migration of ERK1/2 MAP kinases. 

To explain these relationships, it was hypothesized that a physical interaction occurs between TRX-1 and the ERK1/2 MAP kinases under stress conditions. This is predicated on a previously described Cysteine-based heterodimer between TRX-1 and the phosphatase PTEN [20]. *In silico* analysis [21,22] of a hypothetical interaction between TRX-1 and ERK1/2 MAP kinases showed the absence of Cysteine residues on the kinase available for an interaction of this nature (data not shown). To explore other possibilities of interactions between TRX-1 and the ERK1/2 MAP kinases, we performed co-immunoprecipitation experiments. Immunoprecipitation of phospho-ERK1/2 MAP kinases with a rabbit polyclonal antibody from cell lysates of HeLa cells and of HeLa cells expressing PEA-15 treated with H_2_O_2_ for increasing periods was followed by immunoblotting with the same antibody. Western blot analysis showed that the amount of protein immunoprecipitated from cell lysates varied accordingly to the given stimulus. Stripping the blot and re-probing it using a mouse monoclonal anti-TRX-1 antibody showed no results, confirming the absence of interactions between TRX-1 and phospho-ERK1/2 MAP kinases (data not shown). These findings deem that an interaction of the ERK1/2 MAP kinases with TRX-1 is unlikely and suggests that a missing link exists between ERK 1/2 MAP kinases localization/activity and TRX-1 sub cellular compartmentalization under stress conditions. 

### Stress-induced activation and nuclear migration of ERK1/2 MAP kinases regulates the expression levels of Thioredoxin-Interacting Protein – TXNIP

**Figure 4:**
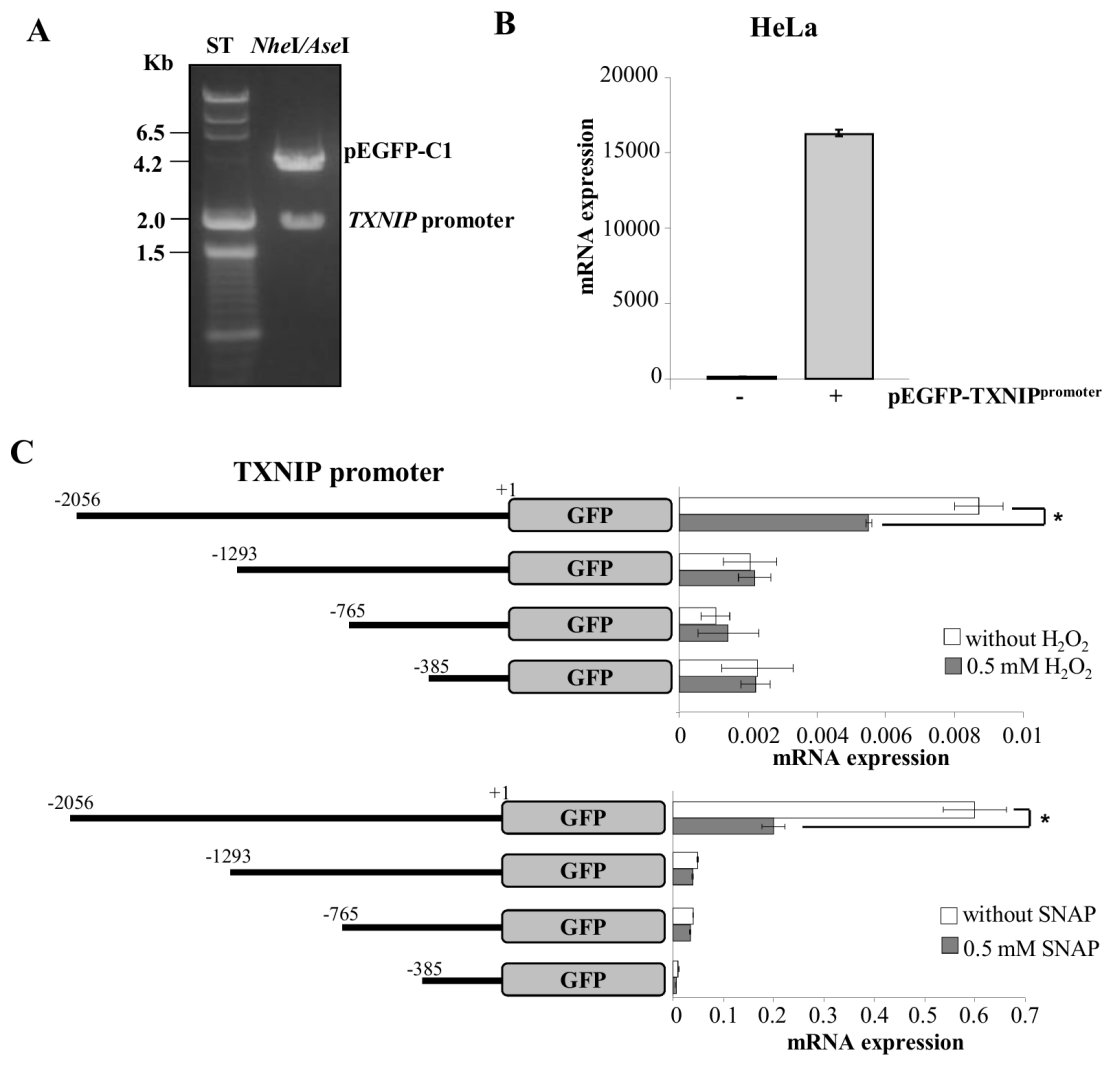
TXNIP promoter construction and regulation of its expression by oxidative and nitrosative stress. (**A**) Scheme representing the molecular cloning of TXNIP’s promoter region replacing the pEGFP-C1 CMV’s promoter region. (**B**) HeLa wild-type cells were transiently transfected with the TXNIP promoter construction and relative levels of GFP mRNA were determined by real-time PCR. (**C**) HeLa cells permanently transfected with the different TXNIP promoter constructs were stimulated with H_2_O_2_ (0.5 mM) or SNAP (0.5 mM). Treatments stimulated strong suppression of GFP expression only in cells transfected with the –2056 base pairs TXNIP promoter construct. Values are reported in the bar graphs and expressed as means ± S.D. (n = 3, *p<0.05).

**Figure 5:**
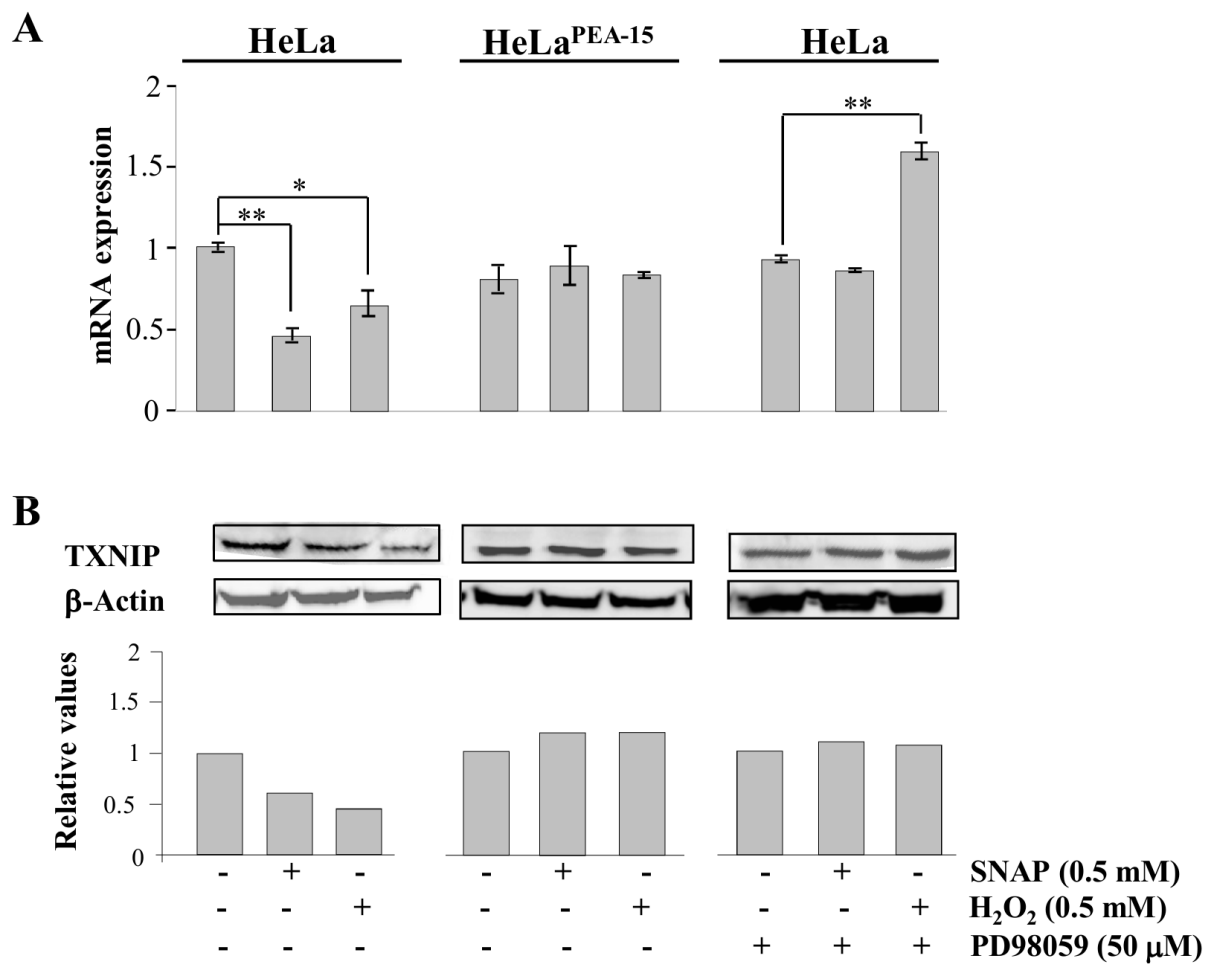
SNAP and H_2_O_2_ -modulated TXNIP mRNA and protein levels in wild-type Hela cells and in HeLa cells expressing PEA-15. (**A**) Relative levels of TXNIP mRNA determined by quantitative real-time PCR in HeLa cells wild-type, in HeLa cells expressing PEA-15, and in HeLa cells wild-type pre-treated with 50μM of PD98059 for 30 min as indicated (see Materials and Methods for details). All cell lines were treated with 0.5mM SNAP or 0.5mM H_2_O_2_ for 2 h at 37°C. Values are reported in the bar graphs and expressed as means ± S.D. (n = 3, *p < 0.05; **p < 0.01). (**B**) wild-type HeLa cells and HeLa cells expressing PEA-15 were incubated with SNAP (0.5 mM) or H_2_O_2_ (0.5 mM) for 2 h at 37°C. Cells were lysed and expression levels of TXNIP were examined by western blotting. Western blot results are representative of four independent experiments. Histogram represents the ratio between the densitometric values of the protein bands corresponding to TXNIP and of β-actin (Lower panel).

TXNIP, which acts as a physiological inhibitor of TRX-1 by forming a mixed disulfide bond with the catalytic active center of TRX-1 [6,22], could be a possible link between TRX-1 and the ERK1/2 MAP kinases nuclear localization under nitrosative/oxidative stress conditions. Expression of TXNIP is down regulated in smooth muscle cells, cardiomyocyte and human lens epithelial cells exposed to high levels of oxidants [9,10,22]. Since the evidence associated with the oxidative-stress-mediated negative regulation of TXNIP expression is circumstantial, we investigated the effects of 0.5 mM H_2_O_2_ and 0.5 mM SNAP on TXNIP promoter activity. 2056 base pairs corresponding to the TXNIP promoter were cloned, as well as three other fragments of 1293, 765, and 385 base pairs, before the ATG initial codon. These sequences were inserted into pEGFP-C1 promoter region, after excising its CMV promoter with AseI and NheI, as previously described [23] (Figure 4A). The TXNIP promoter region was fused to the GFP coding sequence and the number of copies of GFP produced after stimulation is directly related to the level of stimulation of the promoter. This was confirmed by comparing the expression levels of the mRNA for GFP in wild type HeLa cells to that in HeLa cells transfected with the pEGFP-C1_TXNIP promoter construct. Expression was absent in wild type HeLa cells, but was evident in cells transfected with the pEGFP-C1_TXNIP promoter construct (Figure 4B). Transient transfection of HeLa cells with the 2056, 1293, 765, and 385 base pairs fragments of the TXNIP promoter showed that only the cells transfected with the full-length sequence (2056 base pairs) were responsive to oxidative and nitrosative stress conditions. Oxidative and nitrosative stress conditions promoted by exposure of these cells to H_2_O_2_ and SNAP, respectively, led to a decrease on TXNIP promoter activity (Figure 4C). Inhibition of the promoter activity was accompanied by decreased transcript levels of TXNIP in HeLa cells exposed either to 0.5 mM H_2_O_2_ or to 0.5 mM SNAP (Figure 5A – first panel). Over-expression of PEA-15 or treatment of wild type HeLa cells with the MEK inhibitor PD98059 prevented the decrease in transcript levels of TXNIP in cells exposed to stress conditions (Figure 5A – second panel). In cells pre-treated with PD98059 and incubated with 0.5 mM SNAP, transcript levels of TXNIP remained constant as compared to the levels determined for cells not exposed to the nitrosothiol (Figure 5A – third panel). Interestingly, the transcript levels of TXNIP in cells pre-treated with PD98059 and incubated with 0.5 mM H_2_O_2_ were significantly higher as compared to the levels determined for cells not exposed to the oxidant (Figure 5A – third panel). The regulatory pattern for TXNIP expression observed at the mRNA level was reproduced at the protein level with the exception of the condition where cells were pre-treated with PD98059 and incubated with 0.5 mM H_2_O_2_. Western blot analysis of TXNIP expression showed diminished expression levels of the protein in HeLa cells exposed to both stress conditions (Figure 5B – first panel). However, inhibition of phosphorylation and activation of the ERK1/2 MAP kinases or anchoring these MAP kinases in the cytoplasm prevented the stress-mediated inhibition of TXNIP protein expression (Figure 5B – second and third panels). These observations strongly suggest that phosphorylation and nuclear migration of the ERK1/2 MAP kinases are essential events for down regulation of the TXNIP promoter under nitrosative/oxidative stress conditions. 

### The expression levels of TXNIP and the cellular localization of the ERK1/2 MAP kinases regulate stress – induced nuclear migration of TRX-1

**Figure 6:**
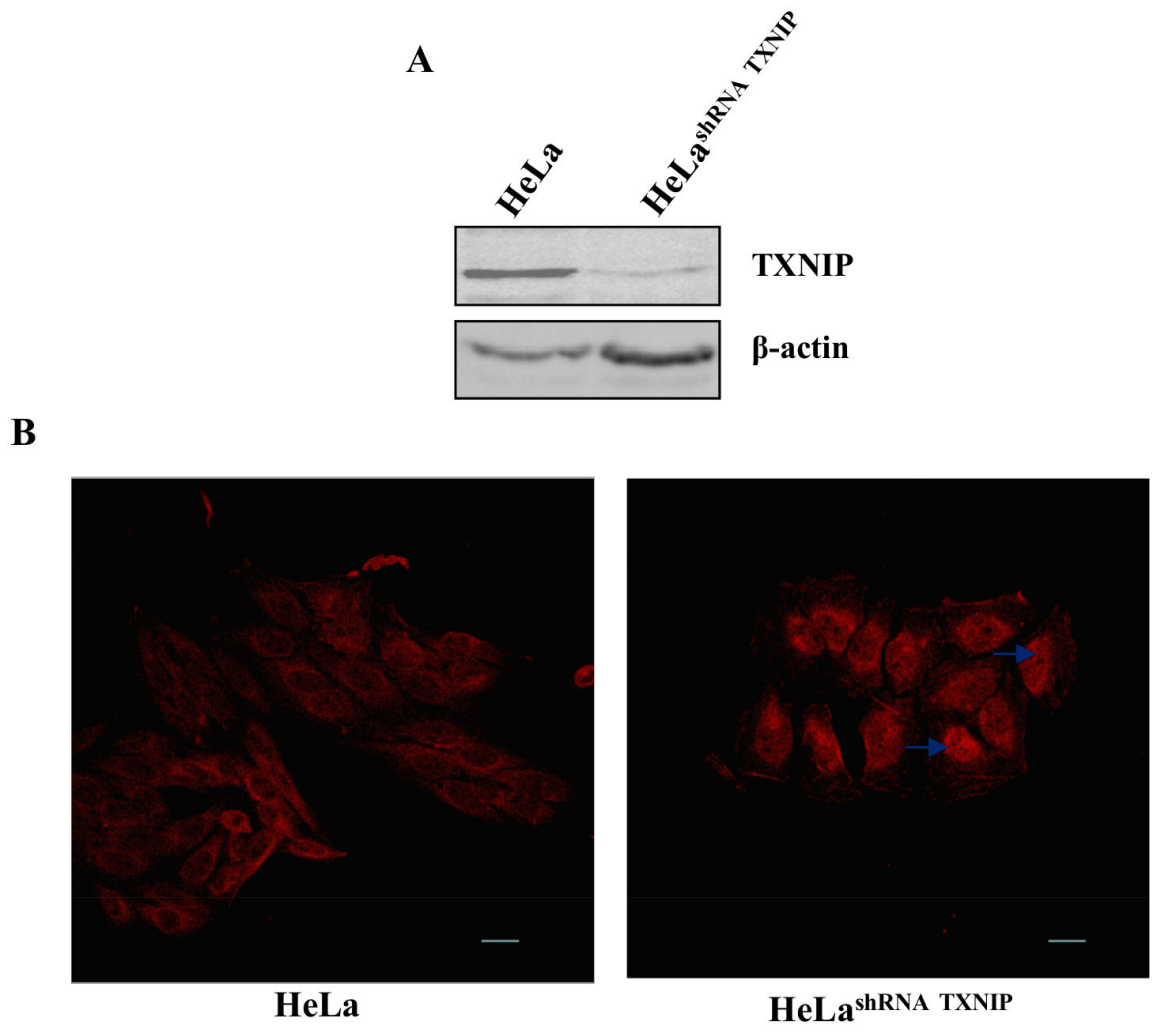
TXNIP silencing and TRX-1 intracellular localization. (**A**) Western blot analysis to determine the effects of mRNA TXNIP knockdown on TXNIP protein levels in HeLa cells permanently transfected with a shRNA for TXNIP. The blot presented is representative of three independent experiments. (**B**) Indirect immunofluorescence to determine TRX-1 sub-cellular localization in HeLa cells permanently transfected with a shRNA for TXNIP. Images shown are representative from three independent experiments. Blue arrows indicate TRX-1 nuclear accumulation. Bar = 20µm.

To demonstrate the importance of the expression levels of TXNIP on the nitrosative/oxidative stress assisted nuclear migration of TRX-1, HeLa cells were permanently transfected with an expression vector encoding the shRNA for TXNIP. Down regulation of TXNIP expression on shRNA-TXNIP permanently transfected HeLa cells was confirmed by determinations of its protein levels (Figure 6A). 

We observed nuclear localization of TRX-1 in shRNA-TXNIP HeLa cells in the absence of stress conditions. Fluorescence was evenly distributed in the nucleus without labeling in the cytoplasm (Figure 6B). TRX-1 remained in the nucleus after exposure of shRNA-TXNIP HeLa cells to 0.5 mM SNAP or 0.5 mM H_2_O_2_ (not shown). 

**Figure 7:**
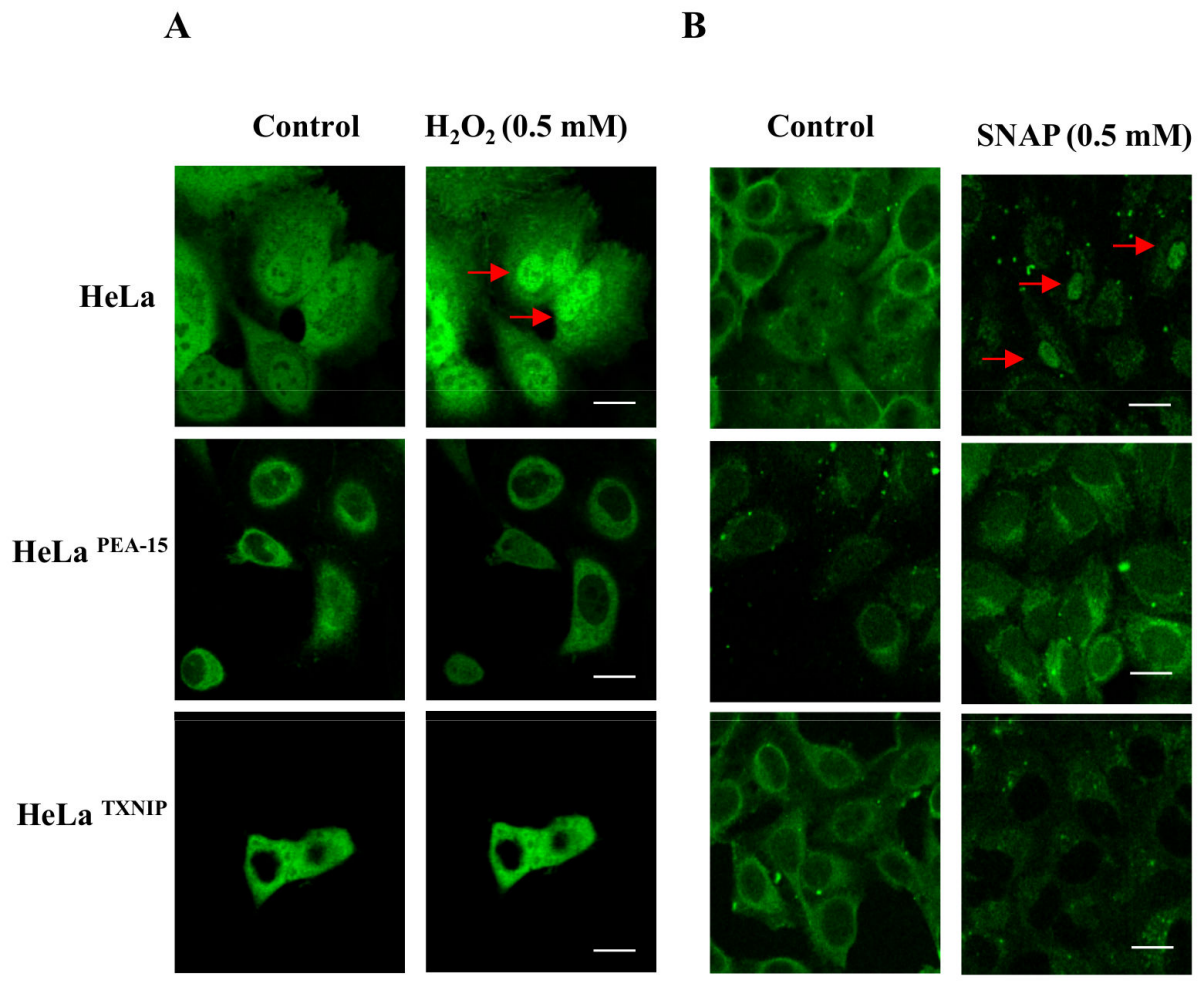
Confocal microscopy based analysis to determine a time-dependent sub-cellular localization of TRX-1 in HeLa cells. (**A**) Wild-type HeLa cells, HeLa cells expressing PEA-15, and HeLa cells expressing TXNIP were plated two days prior to experiment. Transient transfections with pEGFP-C1_TRX-1 were performed on day one. On day two, cells were assayed for TRX-1 sub-cellular localization. Cells were kept in a heated stage chamber (37°C) of the confocal microscope, and a first scanning was recorded to serve as control. After that, media was replaced and H_2_O_2_ was added to a final concentration of 0.5 mM. Subsequent scanning were performed at every 15 min up to 2 h. Bar = 20µm. (**B**) Cells were transfected with pEGFP-C1-TRX-1, treated with SNAP (0.5 mM) for 2 h at 37°C and submitted to indirect immunofluorescence for GFP, to show the localization of TRX-1 in HeLa cells. Red arrows indicate TRX-1 nuclear accumulation.

Using another strategy, to examine the role of TXNIP expression on stress-induced TRX-1 nuclear migration, an expression vector encoding the TXNIP gene was constructed and permanently transfected into HeLa cells. TXNIP or PEA-15 over expressing HeLa cells were transiently transfected with pEGFP-C1_TRX-1. Transient transfection allows for showing the localization of TRX-1 in real time. A strong fluorescent signal associated with TRX-1 expression is seen in the nucleus of pEGFP-C1_TRX-1 HeLa cells after exposure to 0.5 mM H_2_O_2_ (Figure 7A – upper panel). Indirect immunofluorescence was used to detect a strong fluorescent signal associated with TRX-1 located in the nucleus of HeLa cells exposed to 0.5 mM SNAP (Figure 7B – upper panel). Fluorescence was evenly distributed in the cytoplasm without labeling in the nuclear region in PEA-15 or TXNIP over expressing pEGFP-C1_TRX-1 HeLa cells (Figure 7A and B – middle and lower panels, respectively). 

**Figure 8:**
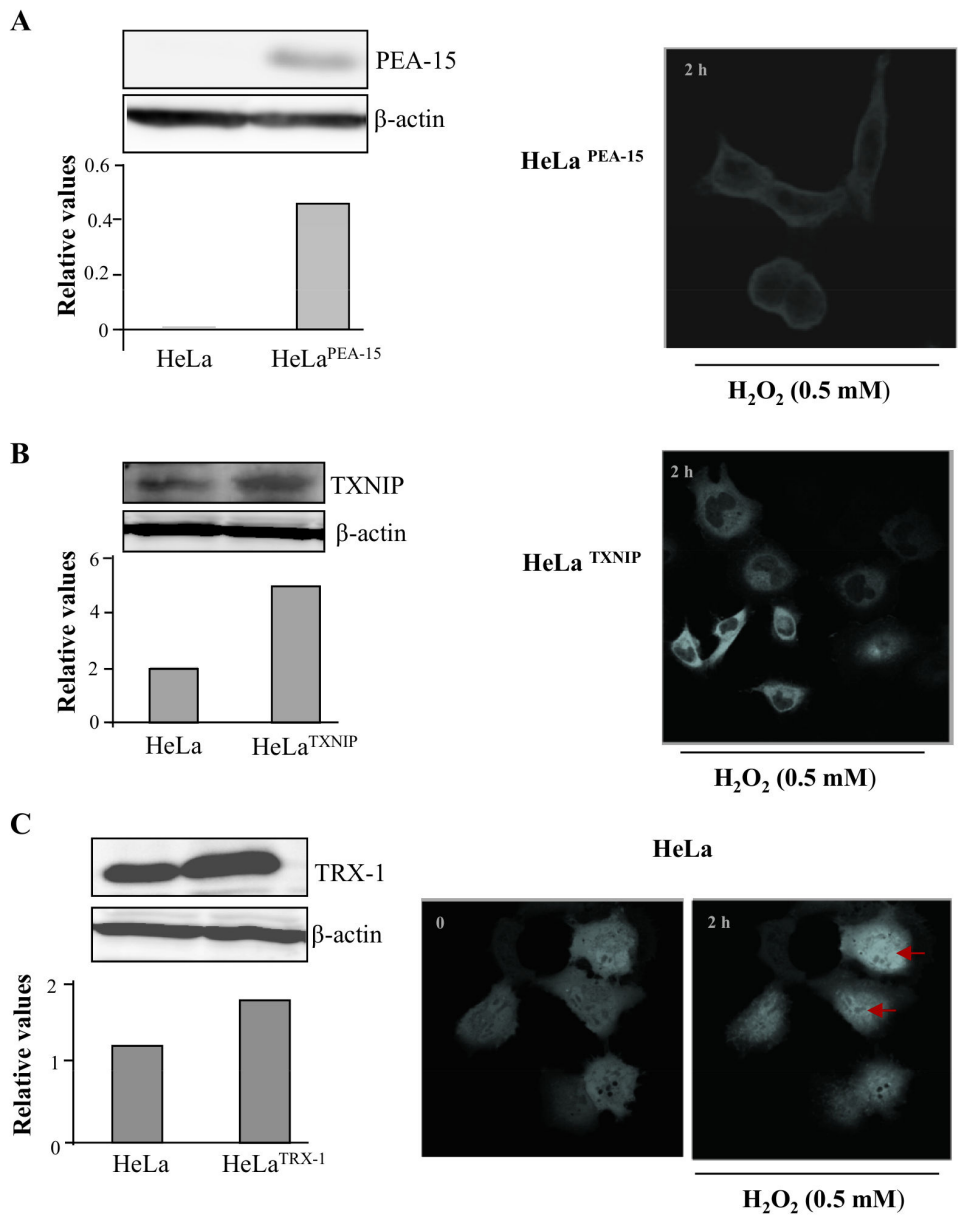
Determination of sub-cellular compartmentalization of TRX-1 in HeLa cells transiently over-expressing PEA-15; TXNIP; TRX-1 and exposed to 0.5 mM H_2_O_2_. HeLa wild-type cells were seeded in complete medium and 24 h later were transiently transfected with the following plasmids pcDNA3_PEA-15, pcDNA3_TXNIP, and pEGFP-C1_TRX-1. After 16 h, transiently transfected cultures were divided to perform either western blot analysis, to confirm over expression of designated inserts, or to perform immunofluorescence-based analysis to determine TRX-1 sub cellular localization after exposure to 0.5 mM H_2_O_2_. (**A**) HeLa cells transfected with pcDNA3_PEA-15. Left panel: western blot analysis of PEA-15 expression. Right panel: Immunofluorescence image of TRX-1 cellular location. (**B**) HeLa cells transfected with pcDNA3_TXNIP. Left panel: western blot analysis of TXNIP expression. Right panel: Immunofluorescence image of TRX-1 cellular location (**C**) HeLa cells transfected with pEGFP-C1_TRX-. Left panel: western blot analysis of TRX-1 expression. Right panel: Immunofluorescence image of TRX-1 cellular location. Red arrows indicate TRX-1 nuclear accumulation.

Permanent transfection of Hela cells with plasmids containing the genes encoding the expression of PEA-15 or TXNIP may lead to phenotypic changes in the transfected cells. To rule out such possibility we transfected Hela cells transiently with the same plasmids. Similar to our findings with the permanently transfected cells, over-expression of PEA-15 or TXNIP prevented TRX-1 nuclear migration stimulated by 0.5 mM H_2_O_2_ Additionally, we transfected transiently HeLa cells with a plasmid containing the gene encoding the expression of TRX-1 and showed nuclear migration of the protein stimulated by 0.5 mM H_2_O_2_ (Figure 8). 

These observations indicate that cellular compartmentalization and the phosphorylation status of the ERK1/2 MAP kinases, and the expression levels of TXNIP, regulate TRX-1 nuclear migration under nitrosative/oxidative stress. 

## Discussion

Cellular compartmentalization of TRX-1 has been associated with specific functions of the protein in the cytoplasm and in the nucleus [2,24]. The redox state of the nuclear compartment is modulated by TRX-1 as part of the regulation of transcription factors [24-26]. TRX-1 is mostly found in the cytoplasm; however, it migrates to the nucleus as a response to elevation of intracellular levels of reactive species [3,4,27]. Previous observations accounted for NGF-induced TRX-1 nuclear migration in rat pre-neuronal PC12 cells. NGF promotes PC12 neuronal differentiation concomitant with release of .NO, produced by an up-regulation of the neuronal isoform of .NO synthase leading to a sustained release of .NO [28]. PD98059 (2-amino-3-methoxyflavone) specific inhibitor of mitogen activated protein kinase kinase (MEK) effectively blocked NGF-induced TRX-1 nuclear translocation and PC12 cells differentiation [4]. Our previous studies focused on the stimulation of the p21Ras-ERK1/2 MAP Kinases signaling pathway by the nitrosothiol SNAP in HeLa cells and its consequences on TRX-1 nuclear migration [3]. Nuclear migration of TRX-1 has been described as part of a survival signaling pathway in response to nitrosative/oxidative pro-apoptotic insults [3,27]. A farnesyltransferase inhibitor of p21Ras inhibited the SNAP-induced TRX-1 nuclear translocation. Downstream to p21Ras, ERK1/2 MAP kinases were activated by SNAP under conditions that promote TRX-1 nuclear migration. PD98059 effectively inhibited SNAP-stimulated ERK1/2 activation and TRX-1 nuclear migration. Although s-nitrosylation of p21Ras triggers a downstream signaling cascade [29-32], its contribution was only partial in sensing the .NO derived from SNAP during TRX-1 nuclear translocation [3]. Inhibition of ERK1/2 MAP kinases prevented SNAP-induced TRX-1 nuclear migration [3]. Studies by Poyssegur et al. [32] showed that activation of ERK1/2 MAP kinases is a process that involves their phosphorylation and changes in their cellular compartmentalization within the cell. In our experimental model, stress-induced TRX-1 nuclear migration is associated with ERK1/2 MAP kinases activation and nuclear migration. Inhibition of MEK, the upstream kinase for ERK1/2 MAP kinases, prevented stress-mediated TRX-1 nuclear translocation. Activated ERK1/2 MAP kinases phosphorylate targets at the cytoplasm and at the plasma membrane level and a fraction of active ERK1/2 migrates to the nucleus by an unknown mechanism [33]. We hypothesized that stress-activated ERK1/2 nuclear migration is accompanied by TRX-1 nuclear migration. Over-expression of PEA-15, a cytoplasmic anchor protein for the ERK1/2 MAP kinases [18] in HeLa cells led to cytoplasmic anchoring of TRX-1 under nitrosative/oxidative stress conditions. Anchoring ERK1/2 MAP kinases in the cytoplasm did not prevent them from becoming phosphorylated upon oxidative stimulus. Over-expression of PEA-15 resulted in higher and sustained levels of phosphorylated ERK1/2 MAP kinases in the absence or in the presence of the oxidative stimulus. It was previously shown that PEA-15 binds to the ERK1/2 MAP kinases and inhibits threonine phosphorylation of fibroblast receptor substrate 2α (FRSα). FRSα is then tyrosine phosphorylated by the fibroblast growth factor receptor, thereby enhancing downstream ERK1/2 MAP kinase signaling [34]. In addition, binding of PEA-15 to the ERK1/2 MAP kinases [18] prevent their nuclear migration and this is a determining factor in stress-induced TRX-1 nuclear migration. 

Binding of TRX-1 to ERK1/2 MAP kinases was ruled out based on *in silico* simulations [20,21]. TRX-1 features the Cysteines 32 and 35 at the active site (1); both residues are able to form disulfide bridges (“half-cystine” cysteine residues) however there were no counterpart “half-cystine” cysteine residues on ERK1/2 MAP kinases. 

An endogenous TRX-1 inhibitor designated as thioredoxin-interacting protein – TXNIP, binds to the active site of TRX-1 and inhibits the activity of TRX-1, thereby modulating the cellular redox state [6,22]. TXNIP prevents TRX-1 nuclear migration resulting in inhibition of the survival signaling pathways mediated by ERK1/2 MAP kinases during cellular exposure to nitrosative stress conditions [25]. TXNIP promotes anti-proliferative effects and rendered vascular smooth muscle cells and NIH3T3 cells more susceptible to oxidative stress and apoptosis [35,36]. 

Regulation of the TXNIP promoter by transcription factors responsive to nutrients has been proposed. In particular, the carbohydrate responsive element and related transcription factors, Max-like protein and MondoA, are positive regulators of the TXNIP promoter [37–41]. Furthermore, the TXNIP promoter is positively regulated by glutamine [42], glutaminolysis, fatty acids and an array of adenosine containing molecules [40,43]. 

However, oxidative and nitrosative stress conditions defined in the present study, are negative regulators of the TXNIP promoter. Inhibition of TXNIP expression levels was observed at the mRNA and protein levels. Our findings corroborate and extend previous observations [9,36]. Furthermore, inhibition of ERK1/2 MAP kinases activities or their cytoplasmic anchorage restores TXNIP expression levels in HeLa cells exposed to nitrosative/oxidative stress conditions. These observations strongly suggest the existence of an ERK1/2-dependent regulatory site on the TXNIP promoter. It also implies that the ERK1/2 MAP kinases survival-signaling pathway operating under stress conditions is a negative regulator of TXNIP expression. 

The PI-3 kinase/Akt pathway, another signaling pathway associated with cell survival has been previously described as a negative regulator of TXNIP expression [12]. Therefore, activation of survival signaling pathways under stress conditions negatively regulates TXNIP expression allowing for TRX-1 nuclear migration and stimulation of cell survival.

## Conclusions

In conclusion, this study shows that nitrosative/oxidative stress-mediated nuclear migration of TRX-1 is associated with intracellular compartmentalization and activation of the ERK 1/2 MAP kinases. It also demonstrates that under nitrosative/oxidative stress conditions, nuclear compartmentalization of ERK1/2 MAP kinases plays a major role in the negative regulation of TXNIP gene expression. In cells exposed to nitrosative/oxidative stress conditions, interactions between TRX-1 and TXNIP coordinated by the ERK1/2 MAP kinases determine the occurrence of TRX-1 nuclear migration and cell survival. These findings may be related to tumor progression. Butler et al. showed that TXNIP gene expression is repressed in many tumor cell lines. Culture of tumor cells with suberoylanilide hydroxamic acid that is an inhibitor of histone deacetylases and an effective inducer of TXNIP expression cause growth suppression and/or apoptosis in these cells [44]. Sheth et al. showed that a mice strain carrying a mutation on TXNIP´s gene developed hepatocellular carcinoma. Approximately 40% of the Txnip-deficient mice developed hepatic tumors as early as 8 months of age [45]. Nishizawa et al. showed that TXNIP expression is down regulated in human bladder carcinoma according to grade and stage. In addition, the authors showed that loss of TXNIP expression facilitates bladder carcinogenesis using a mouse bladder cancer model [46]. Finally, it was recently documented that in A549 human lung cancer cells where TXNIP expression was down-regulated there was a promotion of the epithelial-mesenchymal transition [47]. 

**Figure 9:**
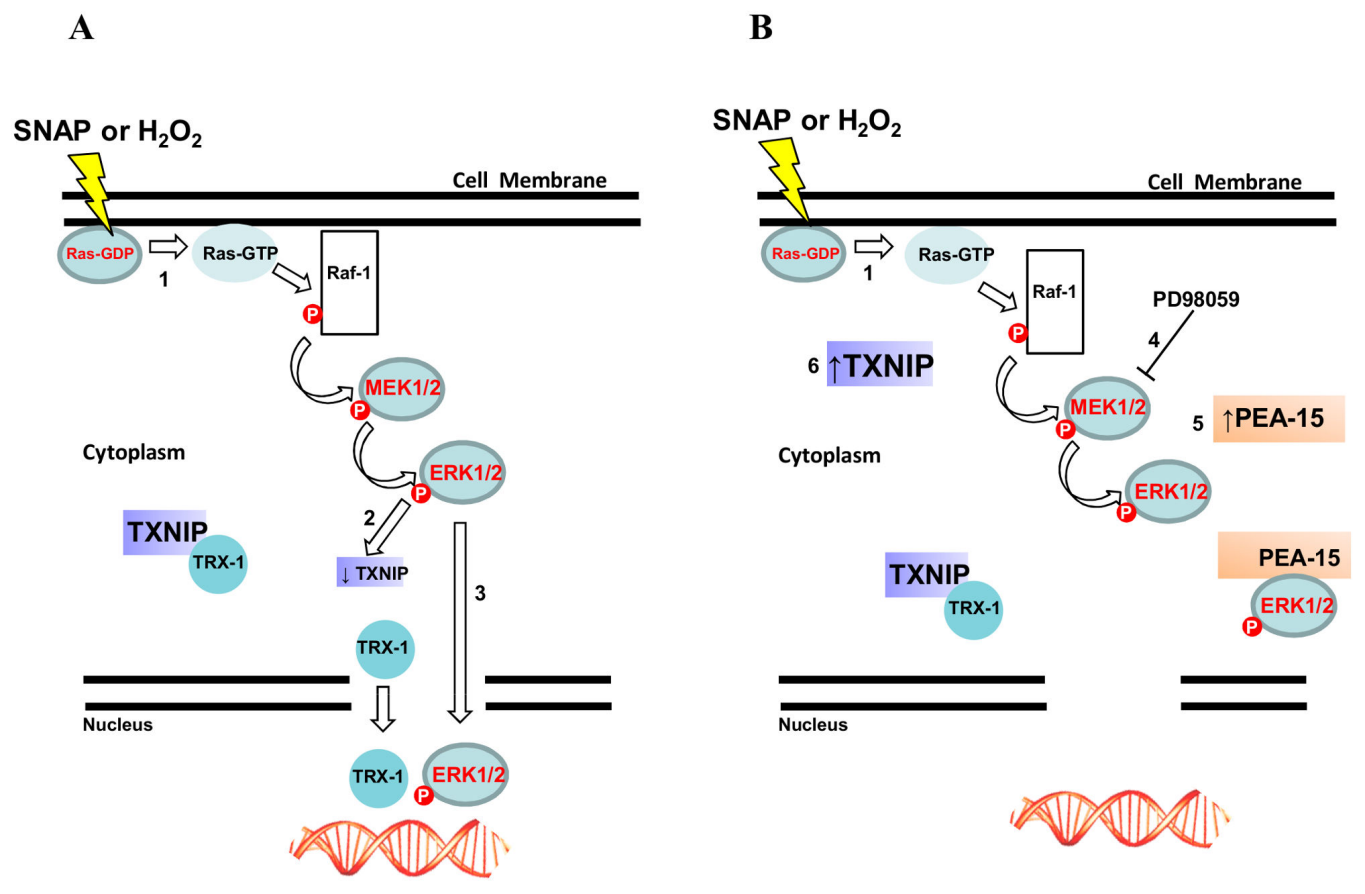
Schematic representation of TRX-1 and the ERK1/2 MAP kinases nuclear translocation stimulated by nitrosative/oxidative stress conditions. (A) The Ras-Raf-MEK-ERK 1/2 signaling pathway is activated in HeLa wild-type cells after exposure to 0.5 mM SNAP or 0.5 mM H_2_O_2_ (1). Under these conditions expression of TXNIP is down-regulated at mRNA and protein levels (2) and TRX-1 and the ERK 1/2 MAP kinases, independently of each other, migrate to the nuclear compartment (3). (B) Nuclear migration of TRX-1 and the ERK 1/2 MAP kinases is prevented in three situations: HeLa cells pre-incubated with the MEK inhibitor PD98059 (4), HeLa cells over-expressing the cytoplasmic anchor of ERK 1/2 MAP kinases – PEA-15 (5), HeLa cells over-expressing the physiological inhibitor of TRX-1, TXNIP (6).

Based on the findings discussed in this study we propose a mechanism where nitrosative/oxidative stress-induced TRX-1 nuclear migration, activation and nuclear translocation of the ERK1/2 MAP kinases, and down regulation of TXNIP expression allow for cell survival. The suggested mechanism is illustrated in Figure 9.

## Supporting Information

Figure S1 **Determination of TRX-1 sub cellular compartmentalization and ERK 1/2 MAP kinases activity in HeLa cells pre-treated with MEK inhibitors.** HeLa wild-type cells were seeded on day one and transfected in the next day with pEGFP-C1_TRX-1. After 16 h cultures were pre-incubated for 30 min with MEK inhibitors PD98059 (50 μM) and UO126 (10 μM). Cultures were further incubated with 0.5 mM H_2_O_2_ for 2 h at 37°C. TRX-1 fused with GFP sub cellular localization was followed in time-lapse confocal microscopy as described in Material and Methods. Cell lysates were subjected to Western blotting with anti-ERK1/2 MAP kinases and anti-phospho-ERK1/2 MAP kinases antibodies. Western blot results are representative of three independent experiments. Histogram represents the ratio between the densitometric values of the protein bands corresponding to the phosphorylated form and of ERK1/2 MAP kinases (Lower panel).
(TIF)
