# Supplementary figures and images for: Nitrosative/Oxidative Stress Conditions Regulate Thioredoxin-Interacting Protein (TXNIP) Expression and Thioredoxin-1 (TRX-1) Nuclear Localization
Source: PLoS One. 2013 Dec 20;8(12):e84588. doi: 10.1371/journal.pone.0084588 (PMC3869934; doi:10.1371/journal.pone.0084588)

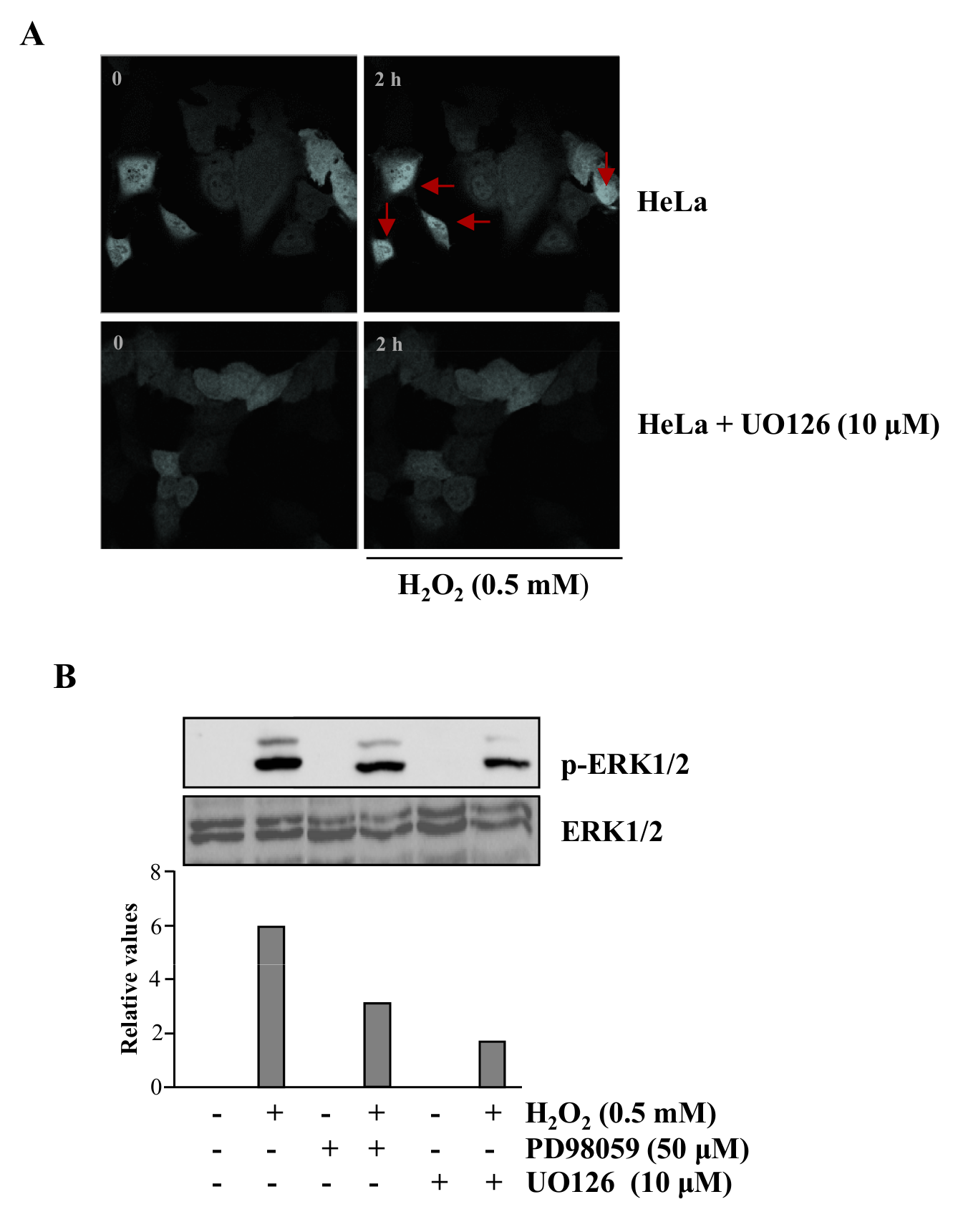

Supplement: Figure S1 — Determination of TRX-1 sub cellular compartmentalization and ERK 1/2 MAP kinases activity in HeLa cells pre-treated with MEK inhibitors. HeLa wild-type cells were seeded on day one and transfected in the next day with pEGFP-C1_TRX-1. After 16 h cultures were pre-incubated for 30 min with MEK inhibitors PD98059 (50 μM) and UO126 (10 μM). Cultures were further incubated with 0.5 mM H2O2 for 2 h at 37°C. TRX-1 fused with GFP sub cellular localization was followed in time-lapse confocal microscopy as described in Material and Methods. Cell lysates were subjected to Western blotting with anti-ERK1/2 MAP kinases and anti-phospho-ERK1/2 MAP kinases antibodies. Western blot results are representative of three independent experiments. Histogram represents the ratio between the densitometric values of the protein bands corresponding to the phosphorylated form and of ERK1/2 MAP kinases (Lower panel). (TIF) [file pone.0084588.s001.tif]
